# Inline Infrared Chemical Identification of Particulate Matter

**DOI:** 10.3390/s20154193

**Published:** 2020-07-28

**Authors:** Javier Núñez, Yunqi Wang, Stefan Bäumer, Arjen Boersma

**Affiliations:** The Netherlands Organisation for Applied Scientific Research, HTC25, 5656AE Eindhoven, The Netherlands; javier.nunez@tno.nl (J.N.); yunqi.wang@tno.nl (Y.W.); stefan.baumer@tno.nl (S.B.)

**Keywords:** particulate matter sensor, inline chemical identification, infrared spectroscopy, hollow waveguide, cyclone, exposome

## Abstract

The health and environmental effects of particulate matter (PM) in the air depend on several parameters. Besides particle size, shape, and concentration, the chemical nature of the PM is also of great importance. State-of-the-art PM sensors only detect the particle size and concentration. Small, low-cost sensors only identify PM according to PM2.5 and PM10 standards. Larger detectors measure the complete particle size distribution. However, the chemical composition of PM is not often assessed. The current paper presents the initial stages of the development of an infrared-based detector for the inline assessment of the chemistry of PM in the air. By combining a mini cyclone that is able to concentrate the particles at least a thousand fold and a hollow waveguide that aligns the flow of particles with infrared light, the feasibility of the concept was shown in this study. A clear differentiation between amorphous and crystalline silica was demonstrated at outdoor PM levels of lower than 1 mg per cubic meter.

## 1. Introduction

The research on exposome is generating more attention in recent years. According to the National Institute for Occupational Safety and Health (NIOSH), exposome can be defined as the measure of all the exposures of an individual in a lifetime and how those exposures relate to health. “An individual’s exposure begins before birth and includes insults from environmental and occupational sources. It is important to understand how exposures from our environment, diet, lifestyle, etc. interact with our own unique characteristics such as genetics, physiology, and epigenetics. The impact of all these exposures on our health is the base for the exposome research” [1]. This is a very complex process, but one of the major efforts needed to complete the exposome picture is to quantify the actual exposure in relevant periods. Since the work environment has an important and potentially avoidable impact on someone’s health, it is important to assess the occupational exposure of a person. Next to occupational settings, knowledge about the precise effects of exposures in the environment is important as well. The concept of exposome can help to close this gap in knowledge and enable the development of preventive measures. For many common disorders, such as respiratory and cardiovascular disorders and cancer, exposures in the environment are an important risk factor, in addition to genetic predisposition, and this exposure has been noted as follows: “The total amount of exposure during a person’s life and the reaction of his or her body to it, the exposome, can help us to better understand and to quantify disease burden” [2].

It is generally recognized that exposure to particulate matter (PM) has a detrimental effect on health [3,4]. PM is made up of many different chemical components, resulting in a different effect on an individual’s health, depending on the chemical composition. Many PM sensors and detectors were developed during the last few decades to quantify the amount and size of PM that people are exposed to. These sensors range from very small and low cost [5,6,7] to large and bulky [8]. These sensors and detectors classify PM in sizes such as PM1, PM2.5, and PM10, where the number indicates the maximum size in micrometers. When positioned along road sites, very useful insight into the air quality and the emission of the traffic is generated. In the Netherlands, a nationwide network of PM detectors that can be consulted by the general public has been established [9,10]. Although very valuable for the general environmental sciences, the use of such a sensor network has some limitations for the exposome approach: (1) the exposure data are not personal but an average over several square kilometers; (2) the PM information is only involving size and concentration but not the chemical composition. To mitigate both issues, there is a need for a small sensor that can chemically identify the various PM sources. It was already recognized earlier that the use of sensors is of great importance to define the external exposome [11].

The chemical analysis of PM in the air is generally done by taking samples in the field using particle filters and taking these samples to a laboratory for analysis [12,13,14,15]. By means of mass spectrometry, atomic absorption spectroscopy, X-ray fluorescence, or Fourier transform infrared spectroscopy (FTIR), the chemical composition can be derived. This way of sampling is slow and expensive due to the required manual labor, and results will only be available at the end of the day after analysis in the laboratory. An alternative, inline method is the use of aerosol mass spectrometry (AMS), which enables us to distinguish between different chemical compounds in airborne particles, but this equipment is bulky and expensive, and it is sometimes limited to specific chemical compounds [16]. These analyzers will therefore only be deployed in a limited number of applications. For a broader assessment of PM distributions on occupational sites or even personal exposure assessment, lower cost and smaller (preferably wearable) devices are required. In this paper, we present the first developments of an inline chemical detector for PM in the air. The use of infrared spectroscopy for the chemical fingerprinting of matter is a very interesting tool that combines analysis speed, the potential for multiparameter sensing, and miniaturization. Infrared absorption spectra of materials generally show multiple absorption bands that can be used for the assessment of chemical bonds and composition. However, for this to be realized, several challenges have to be solved. For example, the concentration of PM in the air is generally too low for a direct chemical analysis, and the interaction between matter and light needs to occur with high efficiency. 

In the context of the development of (low-cost) technology for the detection of low concentration airborne particular matter, a portable demonstrator unit was developed to obtain the chemical fingerprint of airborne particles using a small FTIR spectrometer. The IR-based particle sensor comprises a mini cyclone, a hollow waveguide, an air pump, connecting optical fibers, and a handheld FTIR. The airborne particles are continually fed into the mini cyclone and concentrated in a gas stream that can be produced by an air pump. The concentrated air stream is led through the hollow waveguide where the particulate matter can be identified by an FTIR detector. An air flush system is also included to remove residual particles in the hollow waveguide after every measurement. 

In this demonstrator unit, the mini cyclone is designed to optimize its particle collection efficiency, preferably being able to collect particles down to submicron size. Key mini cyclone geometry parameters are evaluated by simulation for their effects on the particle collection performance. Prototypes of the mini cyclone are fabricated by 3D printing. The effect of particle density and flow rate are also investigated. The prototype is demonstrated by assessing airborne silica and calcium carbonate particles.

## 2. Materials and Methods

**Figure 1:**
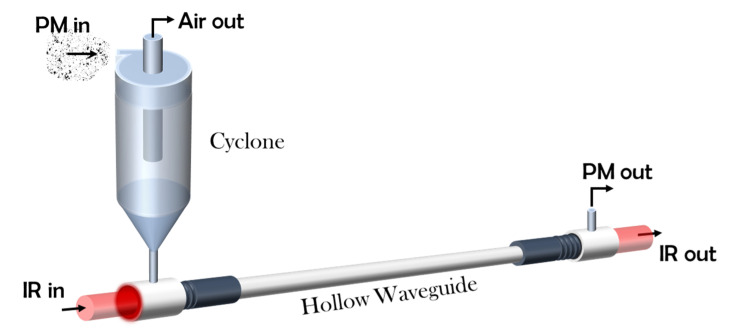
Schematic overview of the particulate matter (PM) chemical identifier (PM-CID).

A schematic drawing of the PM chemical identifier (PM-CID) is shown in Figure 1. The main components of the prototype are the mini cyclone, the hollow waveguide, and the FTIR.

In addition, a vacuum pump is used to maintain a constant flow through the cyclone for the particle separation, as well as some flow controllers to control the flows through the cyclone and the hollow waveguide.

### 2.1. Materials and Hardware

The demonstration of the inline chemical identification was done using several inorganic particles: calcium carbonate (Huber G8 and Q1, Huber Carbonates LCC, Illinois, United States), amorphous silica (Sipernat 570, Evonik, Essen, Germany), and crystalline silica (Quartz and Cristobalite, Merck Sigma Aldrich, Darmstadt, Germany).

The optical particle counter that was used for the assessment of the particle size distribution and concentrations was the TSI OPS3330 (TSI Incorporated, Shoreview, MN, USA), suitable for particle assessment between 0.3 and 10 µm.

Initially, a laboratory FTIR spectrometer was used (Thermofischer, Nicolet 6700, Waltham, MA, USA), equipped with a fiber optic tool to which two SMA coupled fibers can be attached. The FTIR spectra of the pure powders were also measured with this spectrometer by using the attenuated total reflectance (ATR) mode.

For the first prototype of the PM-CID, a commercial small FTIR spectrometer was used. Two options were evaluated: an Agilent 4300 Handheld FTIR (Agilent, United States) and an Arcoptix TE-cooled FTIR-FC (Arcoptix, Switzerland). The specifications of the two spectrometers are slightly different. The Agilent has a spectral range of 4500–650 cm^−1^ with a resolution of 4 cm^−1^ and can be battery operated (DC 15 V). The spectral range of the Arcoptix is 5000–800 cm^−1^ with a resolution of 4 cm^−1^ and needs to be connected to a power socket (AC input adapter 100–240 V, ~1.6 A, 50–60 Hz). The Arcoptix has a direct coupling with optical fibers that can be connected to the hollow waveguide. The Agilent does not have a possibility for direct coupling to fibers. The selection between the two spectrometers depends on their application: the Arcoptix is the preferred option for the optimal connection to optical fibers, whereas the Agilent is essential for measurements with a spectral range below 800 cm^−1^.

The vacuum pump for the air flow through the cyclone and waveguide was a Laboport N86 KT.18 mini diaphragm pump (KNF, Vleuten, Netherlands), having a maximum flow of 5.5 L/min. The flow controllers for controlling the flow through the cyclone and hollow waveguide were obtained from Bronkhorst High Tech (Ruurlo, Netherlands), El-Flow Prestige FG-201CV series (https://www.bronkhorst.com/nl-nl/producten/gas-flow/el-flow-prestige/fg-201cv/).

### 2.2. FTIR Adapter

**Figure 2:**
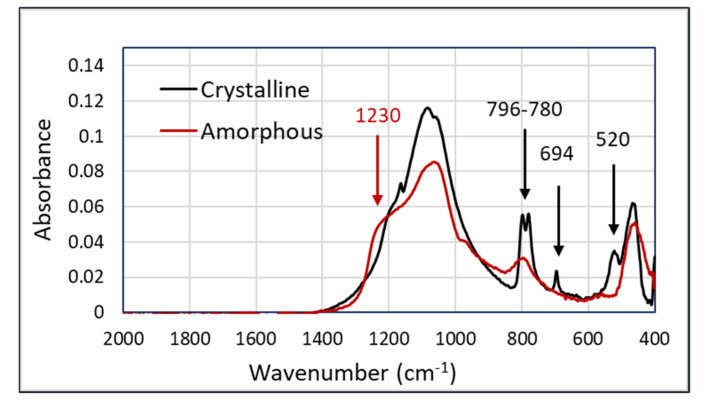
FTIR spectra of amorphous and crystalline silica (both as powders), measured with a Thermofisher Nicolet 6700 on attenuated total reflectance (ATR).

An important use-case for an occupational exposure assessment is the discrimination between crystalline and amorphous silica. The major difference between these two types of silica is found in the peaks at 796–780, 694, and 520 cm^−1^ in the crystalline silica samples. In addition, there is the broadening of the shoulder at 1230 cm^−1^ in amorphous silica. The spectra showing the characteristic features of both components are shown in Figure 2.

**Figure 3:**
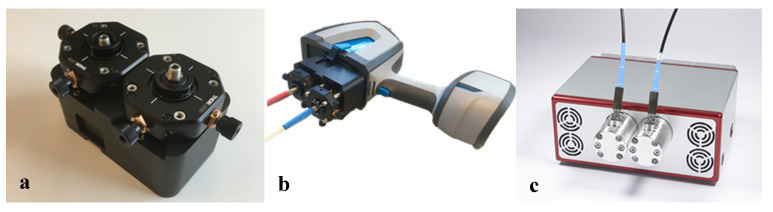
Optical adapter for the Agilent Handheld 4300 FTIR (**a**), assembled to the spectrometer (**b**), and Arcoptix FTIR-FC (**c**).

The spectra in Figure 2, having the most significant peaks below 800 cm^−1^, show that the Arcoptix will be less suitable for the discrimination between amorphous and crystalline silica, although comparing the major peaks at 1100 cm^−1^ will also help us differentiate between amorphous and crystalline silica. Nevertheless, the Agilent was used for the silica experiments. For the connection between the Agilent FTIR and the hollow waveguides, an optical adapter was designed and built. This device is shown in Figure 3a. The optical adapter focuses the mid-infrared light from the FTIR into a SMA multimode fiber (PIR900/1000, polycrystalline AgCl/AgBr fiber, core diameter 860 µm, Artphotonics, Berlin, Germany), and back from the second fiber onto the detector.

In order to keep the connection and transmission losses of the integrated optical adapter, as well as the adapter-fiber connections and the fibers low, all components needed to be well-designed and aligned. The Arcoptix already had a fiber adapter that was optimized to reduce the transmission losses as much as possible.

### 2.3. Mini Cyclone

The suitability of each FTIR spectrometer to detect the presence of chemical substances depends on the sensitivity of the machine and the concentration of the samples. First experiments using the Thermofisher spectrometer revealed that a minimum amount of sample of ca. 0.3 µg is required. Taking the volume of the hollow waveguide of the next section into account (~0.1 mL), the concentration of the PM in the hollow waveguide should be at least 3 µg/mL or 3 g/m^3^. In view of the fact that relevant concentrations of PM in the air range between 10 and 1000 µg/m^3^, the sensitivity of FTIRs is too low for the direct inline chemical identification of PM. This challenge has been approached by the development of a cyclone that can increase the local concentration in the hollow waveguide by several orders of magnitude. The cyclone should be small enough to enable a portable and later on to allow for the development a wearable solution.

**Figure 4:**
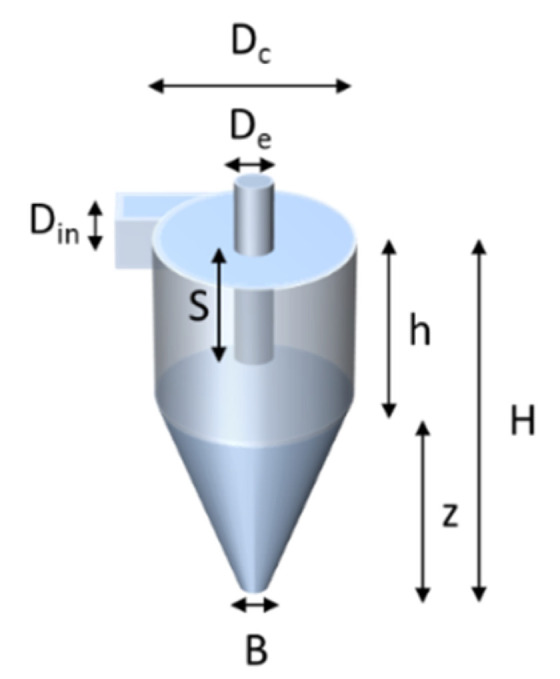
Schematic layout of the mini cyclone.

The design of the cyclone is shown in Figure 4 and was based on the design of Kenny et al. [17,18]. According to these studies, an empirical model was developed, predicting the cyclone cut point as a function of body diameter and flow rate. The experimental data were fitted with models of the following form:

(1) $$\ln\left(D_{50}\right)=a+b\;ln\left(D_{c}\right)-\left(b-1\right)\ln\left(Q\right)$$

where *D_50_* is the penetration cut point in micrometers, *Dc* is the cyclone body inside diameter in cm, *Q* is the flow rate in liters per minute, and *a, b* are empirical constants determined using a nonlinear least squares regression. Both *a* and *b* are constants for a specific cyclone family. A cyclone family is defined as a group of cyclones whose relative dimensions are in fixed proportions to the body diameter. In this study, the cyclone was made based on the design of the extra-sharp-cut cyclone (ESCC) family [18], where the empirical constants are *a* = 0.942 and *b* = 1.846.

According to Kenny’s study, the dimensions *Dc*, *H*, and *S* were shown to have a large influence on *D_5_*_0_. The *D_50_* value is the particle diameter for which 50% is separated and 50% is lost in the main air flow. Hence, the cyclone performance was studied by simulating the influence of the three key dimensions. The flow rate during simulation (*Q*) was 3 L/min. Particle density was 2.65 g/cm^3^ (i.e., silica). The starting point of the values for the cyclone parameters is listed in Table 1. The empirical cyclone efficiencies were compared with the results of numerical modelling using Comsol Multiphysics, simulating the same layout of the cyclone. The traces of the particles were modelled for a number of particle sizes inside the cyclone and the ratio of particles leaving *B* to those entering *D_in_* calculated.

**Figure 5:**
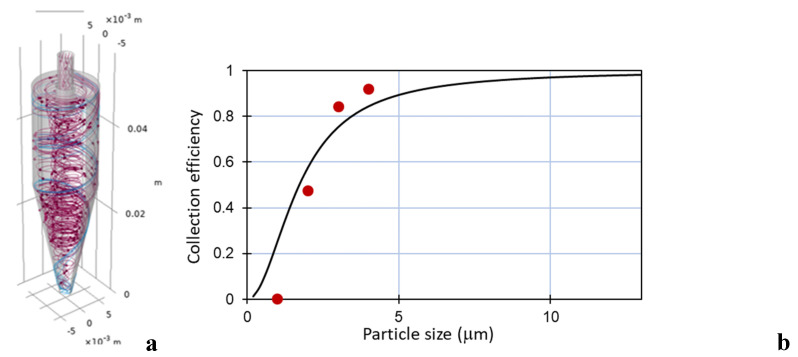
Particle traces inside the cyclone (**a**) and the calculated collection efficiency for the numerical model (dots) and Equation (1) (solid line) (**b**).

The Comsol modelling results (dots) in Figure 5 do not completely match the results of the empirical equation from the literature [18], but the trend is very similar. Apparently, particles smaller than 1 µm are not collected by the cyclone, although the collection efficiency increases very steeply when the particle size increases. The *D_50_* value for the cyclone of Figure 5 is 1.7 µm according to the empirical relation and ca. 2 µm for the numerical model. For environmental and health-related PM measurements, this value is slightly too high: Preferably, >95% of the particles above 0.5 µm should be collected. However, this cyclone was still manufactured and validated for its use in the PM-CID prototype to assess its suitability and demonstrate the concept.

**Figure 6:**
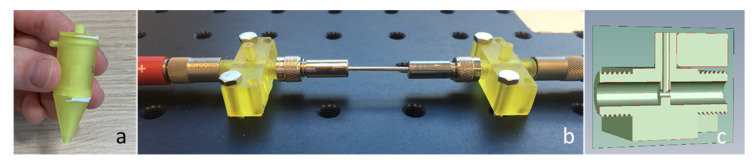
3D printed mini cyclone (**a**), hollow waveguide and two 3D printed adapters (**b**), cross-section of the printed adapters (**c**).

The mini cyclone was manufactured by using 3D printing, by means of a Rapidshape S60 Midi (Rapidshape, Heimsheim, Germany) with a custom formulated acrylic resin [19]. First, the design was converted to a CAD file and used in the layer-by-layer construction of the prototype. The cyclone was printed in separate parts: the bottom cone, the cylinder, and the top in and outlet. Since this is a layer-by-layer deposition process, with layer thicknesses of approx. 50 µm, ridges may be present on the surface of the printed part. This roughness of the surface may influence the flow of the air and possible accumulation of particles in the cyclone. For this reason, a regeneration process was designed, in which the full 3 L/min air flow was led through the cyclone to help remove accumulated particles. The process was validated by the measurement of the weight of the cyclone before and after operation. Depending on the stickiness of the particles, the regeneration time can be adjusted. This process was done manually in the current prototype and was performed for all the experiments showed in this study. An example of the 3D printed cyclone is shown in Figure 6a.

A first estimation of the concentration efficiency can be calculated from flow rates. Assuming a maximum air flow into the cyclone of 5 L/min, an air flow at the bottom exit of 1 mL/min, and a collection efficiency of 80%, the concentration increase is 4000 times. Using the 3 g/m^3^ sensitivity value for the FTIR that was calculated before, the actual airborne particle concentration that should be detectable is now estimated at 750 µg/m^3^.

### 2.4. Hollow Waveguide

Once the particles have been collected in the cyclone, they will be transported to the hollow waveguide by a very small flow of 1–5 mL/min. The hollow waveguide (HWG) is coupled to the FTIR by using two polycrystalline infrared lead fibers of 860 µm in diameter and 1 m in length. The fibers are connected to the waveguide by means of a SMA coupling. Between the hollow waveguide and the fibers, a connector was positioned, also made by means of 3D printing. This connector enables a close connection between the waveguide and fiber, and it allows air (including particles) to enter the hollow waveguide (Figure 6b,c). The gap between the waveguide and fiber should be as small as possible (i.e., <1 mm) to reduce optical losses.

Two types of hollow waveguides were used: one commercial (Polymicro, 1000 µm inner core, Medispec hollow silica waveguide, Molex, United States) and one inhouse manufactured by using a chemical reaction. The procedure to manufacture HWGs inhouse through chemical deposition was performed by using Tollens’ reagent. This was prepared from a 10 mL aqueous solution of AgNO_3_ (0.175 g/10 mL). Around 30 drops of NH_4_OH were slowly added to it; initially the solution gets cloudy and dark, and subsequently clears again (the number of drops may vary and should be added until the solution becomes clear again). Subsequently, 5 mL of KOH solution (0.45 g/10 mL) were added, which again darkens the solution. Finally, NH_4_OH was added until the solution becomes clear once again. Tollens’ reagent must be used on the same day of preparation and never kept stored, as it becomes unstable over time.

An aqueous solution of SnCl_2_ (0.02 g/mL) was first pumped through the 1 mm silica capillary tubes for 5 min, by means of a mini peristaltic pump (Ismatec Reglo) set at 3 mL/min. At this point, the internal surface of the capillary tubes was ready to be coated with silver. For this, the Tollens’ reagent and glucose solution (initial concentration diluted 5 times in deionized (DI) water) were pumped at equal flowrates (3 mL/min) by the peristaltic pump into a T-connector (where both solutions mix), and subsequently into the hollow capillary tubes (where the reaction takes place), with the capillary functioning as a plug flow reactor. As the reagents flow and react through the capillary tubes, a silver layer grows onto their internal surface. This process was run for at least 5 min, when the thickness obtained was enough for allowing maximum internal reflectance. The roughness of the internal Ag coating is dependent on the residence time (conversion) and the reaction rate, which relates to the flowrate through the capillary tubes and the initial concentration of reagents, respectively. For lower reaction rates, more time will be needed for the same thickness, but the quality and reflectance of the coating will be better. For this purpose, the Tollens’ reagent and the glucose solution were diluted again 5 times in DI water before being pumped into the capillary tubes. Finally, an iodine solution in cyclohexane was pumped through the tube to deposit a thin layer of AgI on the surface, for protecting the silver layer and inducing better reflectance.

### 2.5. Integration and Testing

**Figure 7:**
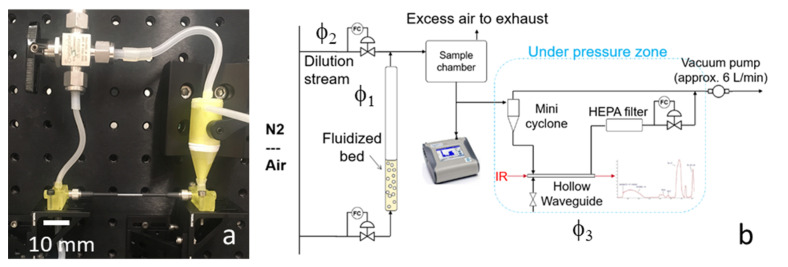
Schematic drawing of the breadboard prototype (**a**) and PM exposure test setup (**b**).

The components presented in the previous sections were integrated into a breadboard prototype. The heart of the prototype is shown in Figure 7a: the hollow waveguide connected to the mini cyclone. A manual valve is also included to switch the flow from the cyclone to the waveguide for regeneration and cleaning.

Figure 7b shows the PM exposure setup, including a fluidized bed to generate the airborne PM and a dilution chamber to regulate the concentration of the PM. Three flows can be controlled independently: φ_1_ through the fluidized bed, φ_2_ through the sample chamber and into the cyclone, and φ_3_ through the HWG. The TSI Optical Particle Sizer is connected to the sample chamber to measure the concentration and particle distribution. A HEPA particle filter is added to prevent particles from entering the vacuum pump. The humidity in the air was low (<30% RH), since nitrogen or compressed air was used as carrier gas.

Several experiments were performed to assess the performance of the PM chemical identifier.

- Three different types of particles: Calcium carbonate, amorphous silica, and crystalline silica (Quartz)
- Various aerosol concentrations: 100 to 2000 µg/m^3^
- Various lengths of the data accumulation: 30 to 300 s

In this stage of the development, no mixtures of particles were assessed. The only aim of the current study is the validation of the concept technology and the sensitivity and selectivity of the FTIR-based approach for PM identification.

A second validation of the prototype was done in an aerosol chamber. The aerosol challenges are done with a BioAerosol Test (BAT) chamber. In this 12 m^3^ chamber (designed by Dycor, Edmonton, Canada), controlled aerosols can be generated either as a continuous or a passing cloud. Both temperature and humidity can be partially controlled. Several wet or dry aerosols can be used. Aerosol concentrations are carefully monitored and measured with reference equipment [20].

## 3. Results

Many experiments were conducted including variations in PM type, particle size and concentrations, and duration of the exposure. In this section we present the most important results and findings of the experiments.

### 3.1. Particle Identification

**Figure 8:**
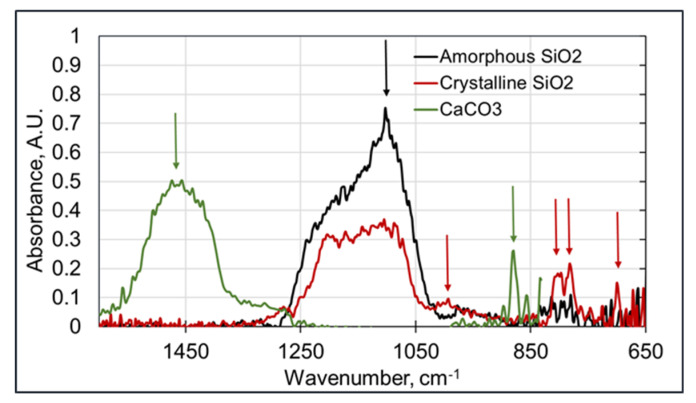
Inline FTIR spectra of amorphous and crystalline silica and calcium carbonate (around 1 mg/m^3^), obtained with the Agilent handheld FTIR (data acquisition of 30 s).

The first and most relevant feature of the PM-CID is the ability to differentiate between various types of particles. Figure 8 shows the inline FTIR spectra during the flow of three types of particles: amorphous silica, crystalline silica, and calcium carbonate. The specific features of the three materials are clearly visible in Figure 8. The peaks at 1100 cm^−1^ corresponding to both forms of silica can be well differentiated from the 1470 cm^−1^ peak of calcium carbonate. In addition, the peaks at 700, 800, and 1000 cm^−1^ are only seen in the crystalline silica spectrum. The shape of the large silica peak around 1100 cm^−1^ is also different for the two silicas. Thus, when assessing the full spectrum, it is well possible to differentiate between various inorganic species. Furthermore, by using suitable data analysis, the composition of the measured particles can be calculated.

### 3.2. Particle Concentrations

There are three airflows in the setup (Figure 7b), φ_1_ (flow through fluidized bed), φ_2_ (flow into the cyclone), and φ_3_ (flow in the hollow waveguide). The particle aerosol generated in the fluidized bed is present in the φ_1_, φ_2_, φ_3_ flows, assuming that all particles are collected by the cyclone and that there are no losses in the system. This means that Ψ_1_ = Ψ_2_ = Ψ_3_, in which Ψ is the particle flow in g/min, and φ the air flow in mL/min.

Ψ_1_ = Ψ_2_ = Ψ_3_ = φ_1_xc_1_ = φ_2_xc_2_ = φ_3_xc_3_ (2)

where c is the concentration of particles in the flow in g/mL.

**Figure 9:**
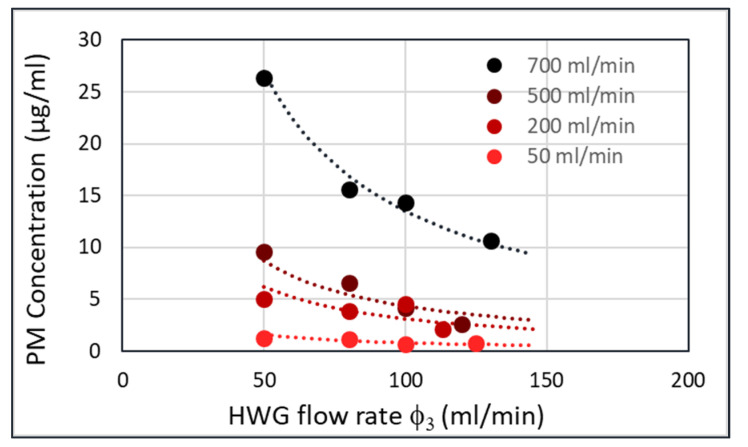
Amorphous silica (Sipernat 570) particle concentration in the hollow waveguide (c_3_) versus flow rate in the cyclone (φ_1_), for four different flow rates in the fluidized bed.

A plot of the particle concentration in the HWG (c_3_) versus the HWG flow rate (φ_3_) yields Figure 9 for four different flow rates of the fluidized bed (φ_1_). This plot was obtained by collecting all particles passing through the HWG in a filter and weighing the filter afterwards. At higher flowrates through the fluidized bed (φ_1_), the PM concentration in the HWG (c_3_) is also higher. However, for each flow rate through the fluidized bed, the product Ψ_1_ = φ_3_xc_3_ should be constant. The lines with the constant Ψ_1_ are shown in the figure as dashed curves. The measured points follow these dashed lines nicely.

**Figure 10:**
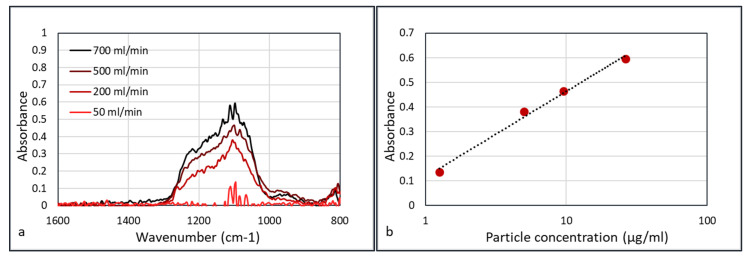
FTIR spectra (Agilent) of the experiments with the HWG flow rate of 50 mL/min and data collection time of 30 s (**a**). Plotting the absorption logarithmically versus concentration, yields a straight line (**b**).

Performing FTIR experiments on all φ_3_ = 50 mL/min flows yields Figure 10.

Figure 10b shows that the absorbance versus concentration plots do not follow the ideal Beer–Lambert law in which absorbance is linear with concentration (A = εcd, where ε is the extinction coefficient, c the concentration, and d the path length). Weingartner et al. [21] discussed the shadowing effect of the particles. With the increasing concentration of particles, the particles absorb a higher fraction of scattered light, which leads to a reduction in optical path. Thus, at a higher concentration of particles, the absorbance is lower than in the ideal Beer–Lambert case, and Weingartner described this with a logarithmic function. This corresponds to the graph in Figure 10b.

### 3.3. Particle Accumulation

**Figure 11:**
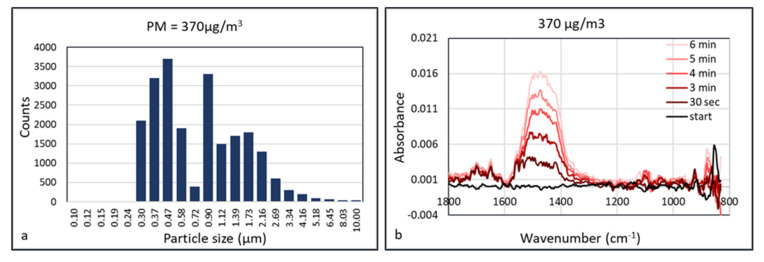
Particle size distribution (**a**) and time dependent FTIR absorption (Arcoptix) (**b**) of calcium carbonate at 370 µg/m^3.^

**Figure 12:**
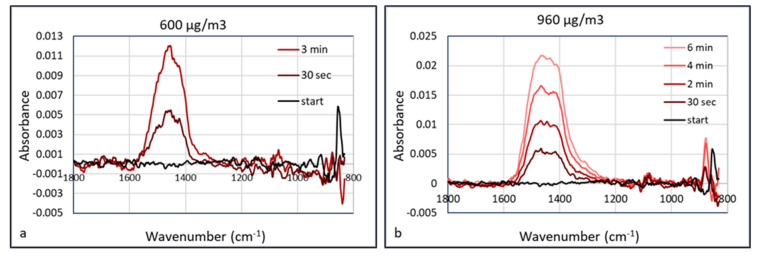
Time dependent FTIR absorption (Arcoptix) of calcium carbonate at 600 µg/m^3^ (**a**) and 960 µg/m^3^ (**b**).

An explanation of the lower “apparent” detection limit of PM in the HWG may be the accumulation of the particles inside the waveguide. Since the flow inside the waveguide is low, particles may accumulate in the capillary without leaving it, as they deposit onto the internal surface. This means that the FTIR spectrum depends on the accumulation or data acquisition time. This was validated using calcium carbonate as an aerosol. Calcium carbonate (Huber G8) aerosols were measured at concentrations of 150, 370, 600, 960, and 2900 μg/m^3^ at a humidity of 50% RH. The aerosol particle size distribution at the concentration of 370 μg/m^3^ is shown in Figure 11. At a low aerosol concentration of 150 μg/m^3^, there was no observable signal shown after collection for 5 min. At a concentration of 370 μg/m^3^, absorbance peak of CaCO_3_ started to show at ~1450 cm^−1^ after collection for 30 s. The signals were enhanced almost linearly as the collection duration increased, which may be the result of more particles being concentrated in the hollow waveguide. The IR spectra measured at aerosol concentrations of 600 and 960 μg/m^3^ are shown in Figure 12.

**Figure 13:**
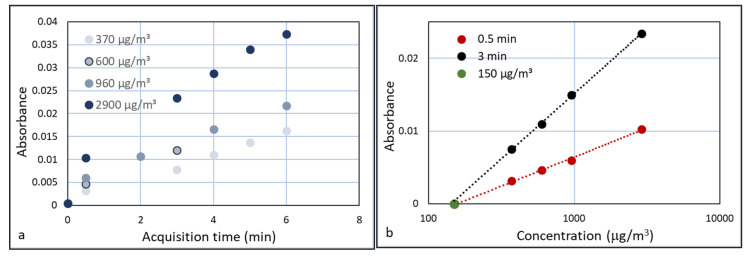
Time dependent FTIR absorption at 1450 cm^−1^ for three concentrations of calcium carbonate (**a**) and a plot of the absorbances at 30 s and 3 min versus concentration ((**b**) on a logarithmic scale).

If, besides the chemistry, the concentration of particles also needs to be measured, a time dependent signal is not useful. To assess the possibilities of the FTIR approach, the height of the absorbances of all experiments and concentrations was plotted versus time. This is shown in Figure 13a. The absorbances of all three concentrations gradually increase with time. When the absorbance was measured at a preselected time (e.g., 30 s or 3 min) and the plotted versus the concentration on a logarithmic scale, Figure 13b is obtained. Both graphs converge to absorbance 0 at around 140 µg/m^3^, which is almost equal to the first measurement at 150 µg/m^3^, which did not show a measurable absorbance (green dot in Figure 13b).

When using this approach, the concentration of the airborne PM can be quantified by calibrating the sensor with standard dust compositions and individual compounds at a specific data acquisition time. When a longer time is taken, the accuracy in the concentration is higher, since the signal-to-noise ratio will be larger. When fast experiments are required, this time can be taken shorter, introducing a larger error.

### 3.4. Crystalline versus Amorphous Silica

**Figure 14:**
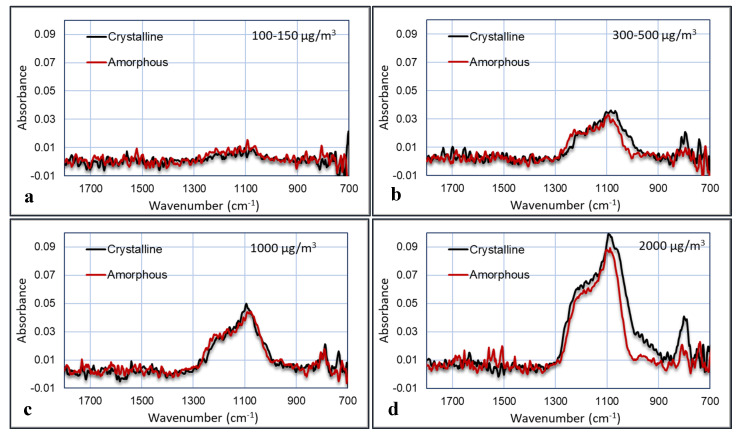
FTIR spectra (with the Agilent) of crystalline versus amorphous silica for four different concentrations in air, using 2 min of data acquisition: 100–150 µg/m^3^ (**a**); 300–500 µg/m^3^ (**b**); 1000 µg/m^3^ (**c**); 2000 µg/m^3^ (**d**).

The last experiment was performed in the bioaerosol test chamber, where the PM was mixed with humidified air. The comparison between amorphous and crystalline silica was done at four concentrations: >2000, ~1000, 300–500, and 100–150 µg/m^3^_._ The concentration of the particles was measured with the TSI OPS3330. The plots of the FTIR absorption versus the wavenumber for the four experiments are shown in Figure 14. The noise in the plots was reduced by averaging the signal over a small wavenumber band. The relative humidity was 50%–60%.

**Figure 15:**
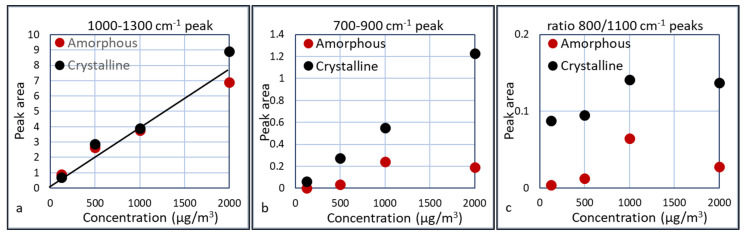
Peak area of the 1000–1300 cm^−1^ (**a**) and the 700–900 cm^−1^ (**b**) peaks and the ratio between the (700–900 cm^−1^)/(1000–1300 cm^−1^) peaks (**c**).

These graphs show that the IR absorbance is directly correlated to the particle concentration. Furthermore, the peak that is characteristic for crystalline silica at 800 cm^−1^ is clearly visible and indeed larger in the crystalline samples than in the amorphous samples, although this is less pronounced in the 1000 µg/m^3^ experiment. Several plots were made in which the area of the peaks are plotted versus the particle concentrations. These areas are calculated as the area below the curves between 1000–1300 cm^−1^ and between 700–900 cm^−1^. The results are shown in Figure 15.

Taking the results of Figure 15a, a linear extrapolation to a measurable peak area gives an estimation for the detection limit of the presence of silica, which is ca. 50 µg/m^3^. For the differentiation between crystalline and amorphous silica, a concentration of at least 200 µg/m^3^ seems required, since the crystalline peak only starts to develop after 200 µg/m^3^. The presence of crystalline silica can be derived from the ratio of the two peaks or from the broader peak of crystalline silica at the 1000 cm^−1^ side. Apparently this ratio is slightly dependent on the silica concentration. This is most likely due to the fact that the 700–900 cm^−1^ peak is small at low concentrations and introduces some error. The 700–900 cm^−1^ peak for the 1000 µg/m^3^ is relatively large compared to the other experiments. A possible explanation is the presence of residual crystalline silica in the cyclone or waveguide, thus enhancing the crystalline peak. This is an indication that we should assess the regeneration of the cyclone and waveguide carefully. The ratio of the two peaks for crystalline silica is ca. 5 times larger than the ratio for amorphous silica. Further experiments need to be done to assess whether this is a constant factor over a larger concentration range.

## 4. Discussion and Conclusions

This paper presents an inline particulate matter chemical identifier prototype. We have shown for the first time (to our knowledge) the real-time chemical assessment of inorganic airborne particles using infrared spectroscopy. Other methods are known, such as aerosol mass spectroscopy (AMS), but these methods measure other properties to calculate chemical composition, and their capabilities are often limited to nonrefractory components. To our knowledge, no discrimination between amorphous and crystalline silica has been achieved by using AMS. In addition, in the development of a smaller and wearable device, the infrared technology appears to be very suitable.

Using a sufficient number of scans, the Agilent and Arcoptix devices can detect absorbances of 0.01 with sufficient signal-to-noise ratio. A linear extrapolation of the line in Figure 10b shows that an absorbance of around 0.01 corresponds to a particle concentration in the HWG of 0.5 µg/mL, and therefore c_3_ = 5 10^−7^ g/mL and φ_3_ = 50 mL/min: Ψ_3_ = 2.5 10^−5^ g/min. Since the volume of the HWG is 8 10^−8^ m^3^, the minimum amount of material in the HWG that can be detected is 0.04 µg. This is approximately 10 time lower than the 0.3 µg that was estimated in the beginning of the paper, using only the FTIR. This is most likely caused by the accumulation of particles in the HWG and the confinement of the light in the HWG, resulting in a better interaction with the particles and therefore a higher efficiency. Since the HWG contains a concentrated stream from the cyclone, this concentration corresponds to a much lower aerosol concentration of the actual air sample.

Regarding the detection limit capabilities of the sensor, the following assumptions have been made: The flow φ_2_ is determined by the vacuum pump at 5000 mL/min, which corresponds to c_2_ = φ_3_xc_3_/φ_2_ = 2.5 10^−5^/5000 = 5 10^−9^ g/mL = 5 mg/m^3^. However, when the HWG flow rate is set to 1 mL/min instead of 50 mL/min, the detection limit decreases to 100 µg/m^3^, which is already sufficient for occupational applications.

The particle concentrations that could be measured in the laboratory tests (≥100 µg/m^3^) were in line with the predicted values from the previous paragraph, based on the FTIR sensitivity, the cyclone concentration behavior, and the flow through the hollow waveguide. A clear differentiation was found between various types of silica and calcium carbonate, i.e., the major constituents of construction dust. This sensor potentially enables the detection of the presence of crystalline silica on construction sites, which allows health, safety, and environment (HSE) officials (from e.g., construction companies) to take action to protect the health of their employees. Critical concentrations for this application range between 50 and 200 µg/m^3^ [22].

It was seen that particles are accumulated in the HWG, which generated a time dependent signal. However, using appropriate calibration protocols and a fixed data acquisition time, concentration measurements may become feasible. The calibration and calculation of the sensor should also include the influence of particle mixtures on the data acquisition protocol. This also means that the detector needs to be regenerated after each data point. So far, this was done manually by switching a valve. In the future demonstrator, this will be automated by using electronically controlled valves. The full air flow capacity of the vacuum pump will be passed through the HWG, removing most of the particles that were accumulated. In addition, surfaces, bends, and angles will be smoothened to reduce accumulation at these positions.

The current detector prototype is still relatively large (approx. 30 × 30 × 30 cm^3^) but could already be deployed as a portable device on working sites. Next to the lab tests, field tests are being planned in the near future. The next demonstrator will be smaller when using a smaller vacuum pump and more miniaturized infrared detection equipment. By optimizing the mini cyclone, connections, and a waveguide, the detection limit is envisioned to decrease to levels below 50 µg/m^3^. This development enables the use of this device in environmental applications, in which PM concentrations between 1 and 100 µg/m^3^ are relevant.

## 5. Patents

Several patent applications have been filed based on the combined cyclone, the hollow waveguide, and the infrared detection method.

## Figures and Tables

**Table 1:** The values of the dimensions of the cyclone of Figure 4.

**Unit (in mm)**	***D_c_***	***D_e_***	***B***	***D_in_***	***S***	***h***	***Z***	***H***
	13.3	3.3	2.67	1.6	5.3	22.2	26.7	48.9
